# Prognostic value of steroid receptors after long-term follow-up of 2257 operable breast cancers.

**DOI:** 10.1038/bjc.1996.291

**Published:** 1996-06

**Authors:** M. F. Pichon, P. Broet, H. Magdelenat, J. C. Delarue, F. Spyratos, J. P. Basuyau, S. Saez, A. Rallet, P. Courriere, R. Millon, B. Asselain

**Affiliations:** Groupe de Biopathologie Tissulaire et Moléculaire, Fédération des Centres de Lutte contre le Cancer, Paris, France.

## Abstract

The prognostic value of oestrogen receptor (ER) and progesterone receptor (PR) was estimated through a multicentric study of 2257 operable breast cancer patients followed up for a median of 8.5 years. None of the patients had received adjuvant therapy. The series included 33.3% stage I patients, 57.1% stage II, 5.7% stage IIIa and 2.4% stage IIIb. At the end point of the study 589 metastases and 537 deaths from cancer were recorded. Receptor measurements were performed by radiolgand assay according to a uniform protocol. A total of 68.8% of the tumous were ER positive and 54.0% PR positive ( > or = 10 fmol mg-1 cytosol protein). In univariate analysis, ER and PR status (positive/negative) were of prognostic value (P < 0.001) for the disease-free interval (DFI), the metastases-free interval (MFI) and the overall survival (OS). The OS of the patients after a first metastasis was also significantly different between ER-positive and -negative tumours (P < 0.001). In multivariate analysis (Cox proportional hazard model, 1665 patients), only the ER status showed a significant difference (P < 0.01) between positive and negative groups regarding the DFI, MFI and OS. By using Cox non-proportional, time-dependent models, we show that the predictive value of ER status of the primary tumour decreases by approximately 20% per year, losing its significance after 8 years of follow-up. Overall, when compared with TNM and histological grading, ER and PR status have a low prognostic value, their major interest remaining solely in the domain of therapeutic decision.


© 1996 Stockton Press All rights reserved 0007-0920/96 $12.00

### Prognostic value of steroid receptors after long-term follow-up of 2257 operable breast cancers

MF Pichon, P Broet, H Magdelenat, JC Delarue, F Spyratos, JP Basuyau, S Saez, A Rallet, P Courriere, R Millon and B Asselain

*Groupe de Biopathologie Tissulaire et Moléculaire, Fédération des Centres de Lutte contre le Cancer, 101 rue de Tolbiac, 75654 Paris Cedex 13, France.*

**Summary** The prognostic value of oestrogen receptor (ER) and progesterone receptor (PR) was estimated through a multicentric study of 2257 operable breast cancer patients followed up for a median of 8.5 years. None of the patients had received adjuvant therapy. The series included 33.3% stage I patients, 57.1% stage II, 5.7% stage IIIa and 2.4% stage IIIb. At the end point of the study 589 metastases and 537 deaths from cancer were recorded. Receptor measurements were performed by radioligand assay according to a uniform protocol. A total of 68.8% of the tumours were ER positive and 54.0% PR positive ($\geqslant$10 fmol mg$^{-1}$ cytosol protein). In univariate analysis, ER and PR status (positive/negative) were of prognostic value ($P<0.001$) for the disease-free interval (DFI), the metastases-free interval (MFI) and the overall survival (OS). The OS of the patients after a first metastasis was also significantly different between ER-positive and -negative tumours ($P<0.001$). In multivariate analysis (Cox proportional hazard model, 1665 patients), only the ER status showed a significant difference ($P<0.01$) between positive and negative groups regarding the DFI, MFI and OS. By using Cox non-proportional, time-dependent models, we show that the predictive value of ER status of the primary tumour decreases by approximately 20% per year, losing its significance after 8 years of follow-up. Overall, when compared with TNM and histological grading, ER and PR status have a low prognostic value, their major interest remaining solely in the domain of therapeutic decision.

**Keywords:** steroid receptors; breast cancer; prognosis

The majority of the studies on steroid receptors and prognosis of early breast cancer have found a positive correlation between the receptor status and patients' outcome.

Several lines of data suggest that the expression of oestrogen and progesterone receptors in the primary tumour is related to the degree of differentiation of the tumour and its proliferation rate. The prognostic value of steroid receptors is probably linked to this relationship.

Nevertheless, many discrepant results have been published relating to the actual prognostic value of steroid receptors. Several reports show that, with increasing follow-up, the initial advantage of positive receptor assays in terms of disease-free interval and survival vanishes (review in Raemaekers *et al.*, 1985).

To address this question we set up a multicentric study of patients diagnosed and treated for primary breast cancer from the beginning of the 1970s. None of the patients had received adjuvant therapy, and receptor assays were all performed using radioligand techniques according to French national protocol (Martin *et al.*, 1981).

### Patients and methods

### *Patients*

The 2257 patients were diagnosed and treated between the beginning of 1974 and the end of 1984 at eight different cancer centres, and the results of their follow-up were studied up to September 1993. The median follow-up time was 8.5 years. At 5 years 7.7% of the patients were lost to follow-up. Bilateral cancers and patients with any kind of adjuvant therapy were excluded from the study.

Information was recorded for each patient including age and hormonal status at diagnosis, clinical stage, histological classification and grade, pathological size and axillary lymph nodes status, steroid receptor status, as well as primary treatment (Table I).

The mean age of the patients was 59 years (range 20-98 years) and 74 patients (3.3%) were under 35 years. Sixty-five per cent of the patients were post-menopausal (range between centres 14-44%). Significant differences ($P<0.0001$) in the mean ages (range 55-65 years) and in the proportion of menopausal patients (range 14-44%) were also observed between centres.

Staging of the disease was performed according to the UICC criteria (stage I, 33.3%; stage II, 57.1%; stage IIIa, 5.7% and 2.4% stage IIIb) (Table I). Significant differences ($P<0.0001$) in the proportions of the different stages were also observed between centres (range 13-55% for stage I and 44-68% for stage II). The primary treatment was tumorectomy (32.7%) or mastectomy with axillary dissection (67.3%), followed by radiotherapy for 49.4% of cases.

Complete follow-up histories of the patients were recorded. During the first year, the follow-up was conducted at 3 month intervals, then over the following 2 year period at 6 month intervals, and thereafter annually.

### *Methods*

*Histology* Tumour size was recorded as the maximum diameter of the surgically removed tumour mass. Axillary lymph nodes status (mean number examined: 13) was assessed by histological examination for 2045 patients. Grading of the tumours was performed according to the Scarff, Bloom and Richardson method (SBR) (Bloom and Richardson, 1957).

*Steroid receptors assays* Cytosol oestrogen (ER) and progesterone (PR) receptors were measured by the dextran-coated charcoal assay with tritiated ligands (Martin *et al.*, 1981). Before the development of a national quality control in 1981, subsequently linked to the EORTC (European Organization for Research and Treatment of Cancer) quality control, all participants used internal quality controls made

Correspondence: M-F Pichon, Laboratoire d'Immunoanalyse, Centre René Huguenin, 35 rue Dailly, 92210 Saint-Cloud, France
Received 21 July 1995; 7 November 1995; accepted 9 January 1996

**Table I** Population characteristics

| *Characteristics* | *Number (%) of patients* | |
|---|---|---|
| Age | | |
| Mean (years) | 59 | |
| Range (min, max) | 20–98 | |
| Hormonal status | | |
| Premenopausal | 791 | (35.0) |
| Post-menopausal | 1466 | (65.0) |
| TNM stage | | |
| I | 752 | (33.3) |
| II | 1290 | (57.1) |
| IIIa | 130 | (5.7) |
| IIIb | 56 | (2.4) |
| ND | 29 | (1.5) |
| Tumour size (mm) | | |
| <20 | 964 | (42.7) |
| 20 to 50 | 1038 | (46.0) |
| ⩾50 | 163 | (7.2) |
| ND | 92 | (4.1) |
| Histology | | |
| Ductal | 1849 | (82.0) |
| Lobular | 105 | (4.6) |
| Other histology | 303 | (13.4) |
| Histological grade (SBR) | | |
| I | 466 | (20.6) |
| II | 1144 | (50.7) |
| III | 402 | (17.8) |
| ND | 245 | (10.9) |
| Axillary lymph nodes (pN) | | |
| None | 1511 | (66.9) |
| 1–3 | 443 | (19.6) |
| ⩾4 | 91 | (4.0) |
| ND | 212 | (9.5) |
| ER | | |
| Negative | 571 | (25.3) |
| Positive | 1554 | (68.8) |
| ND | 132 | (5.9) |
| PR | | |
| Negative | 948 | (42.0) |
| Positive | 1218 | (54.0) |
| ND | 91 | (4.0) |

ND, not determined; ER, oestrogen receptors; PR, progesterone receptors. Positivity if ⩾10 fmol $mg^{-1}$ cytosol protein.

of powders from rabbit or rat uterine tissues kept in liquid nitrogen. Comparisons of the results from exchanged series of tumour tissues were also performed by the members of the group. The cut-off level for receptor positivity was 10 fmol $mg^{-1}$ cytosol protein for both receptors.

*Statistical methods* Comparisons between steroid receptors and clinical and histological data were performed by using standard $\chi^2$ tests in the relevant contingency tables. Two-sided *P*-values under 0.05 were considered significant. Disease-related deaths were scored as an event with censoring of the other patients for non-cancer-related death at the time of last follow-up. The disease-free interval was calculated from the date of the first treatment. First recurrence or first metastasis were scored as an event, censoring the other patients at the time of the last follow-up or of death. Local recurrence was defined as tumour arising in the treated breast or as a nodal recurrence. Survival curves were derived from Kaplan–Meier estimates (Kaplan and Meier, 1958). Survival rates are presented with their standard deviation (±s.d.). The prognostic value of ER or PR was tested using the log-rank test (Peto and Peto, 1972), with an estimation of their unadjusted risk ratio.

The influence of ER and PR on prognosis, adjusted for the other prognostic factors, was assessed in a multivariate analysis by the Cox proportional hazard regression model in a forward stepwise procedure (Cox, 1972). The survival time of each patient was assumed to be following its own hazard function expressed as $h(t)=h_0(t)$ exp (**bZ**) where $h_0(t)$ is an unknown hazard function, **Z** the vector of measured values of the covariates for the patient, and **b** the vector of regression coefficients associated with these covariates. The **b** coefficients produced by the analysis show how much each factor contributes to the hazard. Complete data for all clinical and biological variables were available for 1665 out of 2257 patients. Therefore, the Cox model used this subset of 1665 patients. Confounding variables with $k$ subgroups were coded to $(k-1)$ dummy variables. This model assumes a log-linear relation of relative risks between two subsequent subgroups when $k>2$. The relative risks (RRs) are presented with their confidence interval (CI). The classical Cox proportional hazard model implies that the effect of ER concentration is constant throughout the patient's survival experience. To take into account possible violations of this assumption, a separate approach was used by modelling a time-dependent relation between hazard and covariates (Cox model with time-dependent covariates, amended by Kalbfleish and Prentice, 1980). To accommodate this time dependency, receptor status was entered as a time-dependent covariate in the amended model $h(t)=h_0(t)$. exp ([**b**′+**b**″ f($t$)]**Z**) with two functions of time: $f(t)$ = time and $f(t)$ = Ln (time). Annual relative risks, calculated by using the time-dependent hazard function are presented on the same figure as the model curves. All statistical analyses were performed by using BMDP programs.

### Results

### *Histopathology and receptors*

The mean size of the tumours was 28.9 mm (range between centres 20.4–35.6 mm). A total of 1511 (66.9%) patients were node negative, 443 (19.6%) had 1–3 invaded nodes, 91 (4.0%) more than four invaded nodes (range between centres for node-negative patients 42–100%). Eighty-two per cent were ductal carcinomas, 4.6% were lobular carcinomas, the other particular histological types were recorded as a whole group (13.4%). In all 20.6% were SBR grade I (range between centres 14–53%), 50.7% were SBR grade II (range 21–70%), 17.8% were SBR grade III (range 9–36%) (Table I). Significant variations in the mean tumour size, proportions of positive or negative lymph nodes and in the three SBR groups were observed between different centres ($P<0.001$).

Some 68.8% of the tumours were ER positive (mean concentration 93 fmol $mg^{-1}$ cytosol protein), 54.0% were PR positive (mean concentration 73 fmol $mg^{-1}$ protein). For 223 cases only one receptor could be measured because of insufficient tumour tissue. Positivity for both receptors was observed in 53.3% of the tumours, 23.9% were all negative, 19.8% were only ER positive and 3.0% only PR positive. Table II summarises the results of receptor assays from the participating centres.

### *Receptor status and patients characteristics (Table III).*

ER was significantly related to: age ($P=0.005$), menopausal status ($P=0.005$), lymph node involvement ($P=0.03$), tumour size ($P=0.04$), histological type of the tumour ($P=0.0001$) and SBR grade ($P=0.001$). PR was significantly related to clinical stage ($P=0.001$), lymph node involvement ($P=0.015$), tumour size ($P=0.04$), histological type ($P=0.0001$) and grade ($P=0.001$).

### *Patients' outcome*

At the end point of our study, we have recorded 307 local recurrences, 589 metastases and 673 deaths including 537 from cancer. The patients' population also displayed 199 second cancers, including 105 cancers of the contralateral breast. Overall survival at 60 months was 83.8±1.5% and 67.2±1.9% at 120 months.

*Univariate analysis of disease-free interval and of overall survival* A total of 74.1±0.9% of the patients were free of

**Table II** Oestrogen and progesterone receptors according to the participating centres

| | | ER | | | PR | | |
|---|---|---|---|---|---|---|---|
| *Centre* | n | *Median* | *Mean (s.e.m.)* | *Per cent ER+ (CI)* | *Median* | *Mean (s.e.m.)* | *Per cent PR+ (CI)* |
| 1 | 693 | 57.0 | 105.2 (5.7) | 76 (76–82) | 17.0 | 81.0 (5.7) | 59 (55–63) |
| 2 | 608 | 30.0 | 75.9 (5.1) | 76 (73–79) | 16.0 | 49.1 (3.7) | 65 (61–69) |
| 3 | 299 | 81.0 | 141.9 (9.5) | 84 (80–88) | 44.0 | 95.5 (8.1) | 59 (53–65) |
| 4 | 289 | 11.5 | 46.7 (6.6) | 54 (48–60) | 7.0 | 19.7 (2.7) | 38 (32–44) |
| 5 | 229 | 25.0 | 75.0 (8.5) | 64 (58–70) | 11.0 | 124.2 (15.1) | 40 (34–46) |
| 6 | 47 | 48.0 | 110.7 (25.5) | 75 (40–95) | 41.0 | 99.7 (24.3) | 62 (26–88) |
| 7 | 64 | 34.0 | 176.1 (36.7) | 70 (58–82) | 21.0 | 96.7 (23.9) | 56 (44–68) |
| 8 | 28 | 12.5 | 71.6 (31.2) | 46 (27–65) | 51.5 | 141.5 (34.8) | 57 (39–75) |

ER, oestrogen receptors; PR, progesterone receptors. Positivity if $\geqslant$10 fmol mg$^{-1}$ cytosol protein. CI, confidence interval at 95%; s.e.m., standard error of the mean.

**Table III** Correlation between steroid receptors and clinical or histological characteristics

| *Patients* | *ER−* n (%) | *ER+* n (%) | P-value ($\chi^2$) | *PR−* n (%) | *PR+* n (%) | P-value ($\chi^2$) |
|---|---|---|---|---|---|---|
| Age (years) | | | | | | |
| <35 | 28 (42) | 39 (58) | | 38 (53) | 34 (47) | |
| $\geqslant$35 | 540 (26) | 1506 (74) | 0.005 | 903 (43) | 1179 (57) | 0.12 |
| Hormonal status | | | | | | |
| Premenopausal | 226 (31) | 512 (69) | | 287 (37) | 487 (63) | |
| Post-menopausal | 343 (25) | 1034 (71) | 0.005 | 655 (47) | 728 (53) | 0.0001 |
| TNM stage | | | | | | |
| I | 186 (26) | 518 (74) | | 285 (39) | 448 (61) | |
| II | 324 (26) | 901 (74) | 0.5 | 561 (45) | 677 (55) | 0.001 |
| III | 54 (30) | 123 (70) | | 92 (52) | 83 (48) | |
| Tumour size (mm) | | | | | | |
| <20 | 221 (24) | 683 (76) | | 370 (40) | 559 (60) | |
| 20–50 | 251 (36) | 724 (74) | 0.04 | 451 (45) | 547 (55) | 0.036 |
| $\geqslant$50 | 55 (34) | 106 (66) | | 74 (46) | 85 (54) | |
| Histology | | | | | | |
| Ductal | 444 (25) | 1310 (75) | | 731 (41) | 1055 (59) | |
| Lobular | 18 (19) | 74 (81) | 0.0001 | 44 (46) | 51 (54) | 0.0001 |
| Other | 107 (39) | 168 (61) | | 170 (60) | 112 (40) | |
| Histological grade SBR | | | | | | |
| I | 61 (14) | 374 (86) | | 135 (30) | 320 (70) | |
| II | 236 (22) | 843 (78) | 0.001 | 425 (38) | 682 (62) | 0.001 |
| III | 175 (46) | 207 (54) | | 255 (65) | 138 (35) | |
| Axillary lymph node (pN) | | | | | | |
| None | 385 (27) | 1057 (73) | | 652 (44) | 813 (56) | |
| 1–3 | 96 (23) | 318 (77) | 0.03 | 161 (39) | 255 (61) | 0.015 |
| $\geqslant$4 | 35 (36) | 62 (64) | | 50 (54) | 43 (46) | |

ER, oestrogen receptors; PR, progesterone receptors. Positivity if $\geqslant$ 10 fmol mg$^{-1}$ cytosol protein. $\chi^2$, Chi-square test.

disease at 60 months and 64.0±1.1% at 120 months. Some 89.5±0.7% of the patients were free of local recurrences at 60 months, and 83.9±0.9% at 120 months. Overall, 80.0±0.9% of the patients were metastases free at 60 months, 71.4±1.0% at 120 months. The probabilities of survival according to receptor status in the primary tumour are given in Figures I (ER), 2 (PR), and 3 (combined ER and PR). At 60 months (Table IV), the overall survival was 90.1% for ER-positive tumours, 91.5% for PR-positive tumours and 91.4% when both receptors were positive. All the differences between positive and negative results are significant ($P$<0.01). Significant differences were also obtained when comparing receptor status and the disease-free interval or the occurrence of metastases (Figures 4 and 5). The relative risks calculated for ER-negative tumours were generally slightly higher than the corresponding ones for PR. For example, the relative risks of death were 1.75 for ER-negative tumours and 1.68 for PR-negative tumours.

**Figure 1** Overall survival by ER status (-■-, ER$^-$; -□-, ER$^-$). 1261/422 patients ER$^-$/ER$^-$ at risk for 60 months, 482/164 patients ER$^-$/ER$^-$ at risk for 120 months.

*Survival after the first metastasis* We also sought a potential predictive value of steroid receptors after a first metastasis. The univariate analysis shows that both ER and PR have a favourable influence upon survival after a first metastasis, the difference between positive and negative groups being significant at a level of $P$<0.0001 for each comparison (only the ER chart is presented; Figure 6). The median of overall survival after a first metastasis is 29 months for ER-

positive and 15 months for ER-negative tumours (RR = 2.0), and 33 and 16 months for PR-positive and-negative tumours respectively (RR = 1.90).

*Multivariate analysis* The following variables were introduced in the Cox model: age, menopausal status, clinical stage, size of the tumour, node involvement, histological type and grade of the tumour and steroid receptors. We have chosen a cut-off of 35 years for the age groups, in accordance with the results of several studies (Adami *et al.*, 1986; Host and Lund, 1986), showing that the differences in overall survival are at a maximum when using that cut-off. The influence of these factors on disease-free interval, occurrence of metastases and survival was evaluated in a forward stepwise regression. The results for the three models are presented in Table V. For each factor, the entry step number,

**Figure 2** Overall survival by PR status (-■-, PR⁻; -□-, PR⁺). 1017/680 patients PR⁺/PR⁻ at risk for 60 months, 351/285 patients PR⁺/PR⁻ at risk for 120 months.

**Figure 4** Metastasis-free survival according to ER status (-■-, ER⁻; -□-, ER⁺). 1213/369 ER⁺/ER⁻ at risk for 60 months, 436/155 patients ER⁺/ER⁻ at risk for 120 months.

**Figure 3** Overall survival by combined ER and PR status (-□-, ER⁻PR⁺; -◆-, ER⁺PR⁺; -■-, ER⁻PR⁻; -△-, ER⁺PR⁻). 922/340/311/51 patients ER⁺ PR⁺/ER⁻ PR⁻/ER⁺ PR⁻/ER⁻ PR⁺ at risk for 60 months, 321/138/142/20 patients ER⁺ PR⁺/ER⁻ PR⁻/ER⁺ PR⁻/ER⁻ PR⁺ at risk for 120 months.

**Figure 5** Metastasis-free survival according to PR (-■-, PR⁻; -□-, PR⁺). 911/634 PR+/PR− at risk for 60 months, 318/264 patients PR+/PR− at risk for 120 months.

**Table IV** Five and 10 years' results

| | *Survival (%)* | | *Metastasis-free (%)* | | *DF (%)* | |
|---|---|---|---|---|---|---|
| | *5 years* | *10 years* | *5 years* | *10 years* | *5 years* | *10 years* |
| ER* | | | | | | |
| ER⁻ | 75.9 (1.8) | 63.4 (2.2) | 72.1 (2.2) | 65.0 (2.2) | 66.2 (2.0) | 58.0 (2.2) |
| ER⁺ | 90.1 (0.8) | 75.8 (1.3) | 82.2 (1.0) | 73.3 (1.2) | 76.4 (1.1) | 65.8 (1.3) |
| PR* | | | | | | |
| PR⁻ | 80.3 (1.3) | 66.7 (1.7) | 75.9 (1.4) | 67.6 (1.7) | 69.7 (1.5) | 60.6 (1.7) |
| PR⁺ | 91.5 (0.8) | 77.2 (1.4) | 82.9 (1.1) | 74.4 (1.4) | 77.3 (1.2) | 66.3 (1.5) |
| ER and PR** | | | | | | |
| ER⁺PR⁺ | 91.4 (0.9) | 76.7 (1.5) | 82.4 (1.2) | 73.4 (1.5) | 77.1 (1.3) | 66.1 (1.6) |
| ER⁻PR⁺ | 89.0 (4.0) | 75.6 (6.5) | 81.7 (5.0) | 75.9 (6.3) | 71.5 (5.9) | 66.0 (6.7) |
| ER⁺PR⁻ | 87.2 (1.7) | 73.9 (2.5) | 82.0 (1.9) | 72.9 (2.4) | 75.1 (2.2) | 65.4 (2.6) |
| ER⁻PR⁻ | 74.2 (2.0) | 61.6 (2.4) | 70.2 (2.1) | 63.0 (2.3) | 64.9 (2.2) | 55.8 (2.5) |

DF, Disease-free; ER, oestrogen receptors; PR, progesterone receptors. Positivity if ≥ 10 fmol $mg^{-1}$ cytosol protein. * All *P*-values less than 0.01 for the three criteria. ** All *P*-values less than 0.01 testing for heterogeneity and between the group ER⁺PR⁺ and ER⁻PR⁻.

**Figure 6** Survival after a first metastasis according to ER status (-■-, $ER^-$; -□-, $ER^+$). 116/23 $ER^+/ER^-$ at risk for 36 months.

**Figure 7** Relative risk of metastasis according to ER status. Annual observed and calculated risks with a Log–time dependent function (Cox non-proportional hazard model).

the relative risk and its confidence interval are displayed. The disease-free interval is reduced for patients under 35 years, for patients with positive nodes or large tumour size or high SBR grade and when the tumours are ER negative. PR status has no statistical significance regarding the disease-free interval.

The risk of developing metastases increases for patients under 35 years, for positive lymph nodes, for large tumour size, for high SBR grade and for ER-negative tumours. Again, PR status has no prognostic value in this context. With regard to the mortality risk through breast cancer, the significant variables of the Cox model were: menopausal status, high clinical stage, large tumour size, positive lymph nodes, high SBR grade and ER negativity. PR status was found to be non-significant. No significant differences in the relative risks associated to the ER or PR status were found among premenopausal *vs* post-menopausal patients.

**Figure 8** Relative risk of death according to ER status. Annual observed and calculated risks with a time-dependent function (Cox non-proportional hazard model).

**Table V** Adjusted relative risks[a] with their 95% confidence interval (Cox model)

| *Variable* | *Esn* | *Disease-free survival* | *Esn* | *Metastasis-free survival* | *Esn* | *Survival* |
|---|---|---|---|---|---|---|
| Histological grading | 1 | | 1 | | 1 | |
| (SBR) | | 1 | | 1 | | 1 |
| I | | 2.09 (1.61–2.72) | | 2.32 (1.69–3.18) | | 2.45 (1.71–3.50) |
| II | | 2.50 (1.57–3.98)* | | 2.86 (1.67–4.88)* | | 2.80 (1.54–5.07)* |
| III | | | | | | |
| Axillary node status | 2 | | 2 | | 2 | |
| (pN) | | 1 | | 1 | | 1 |
| None | | 1.56 (1.28–1.89) | | 1.85 (1.50–2.31) | | 1.65 (1.30–2.08) |
| 1–3 | | 2.29 (1.36–3.86) | | 2.99 (1.77–5.17) | | 3.23 (1.82–5.69) |
| >3 | | | | | | |
| Tumour size | 3 | | 3 | | 3 | |
| <20 mm | | 1 | | 1 | | 1 |
| 20–50 mm | | 1.46 (1.22–1.74) | | 1.56 (1.27–1.97) | | 1.37 (1.06–1.76) |
| >50 mm | | 2.05 (1.31–3.22) | | 2.06 (1.26–3.38)* | | 1.90 (1.07–3.34) |
| Age (years) | 4 | | 4 | | | |
| >35 | | 1 | | 1 | | |
| ⩽35 | | 2.78 (1.91–4.03) | | 2.78 (1.85–4.17) | | NS |
| ER status | 5 | | 5 | | 4 | |
| Positive | | 1 | | 1 | | 1 |
| Negative | | 1.35 (1.13–1.61) | | 1.45 (1.19–1.75) | | 1.85 (1.55–1.95) |
| TNM stage | | | | | 5 | |
| I | | NS | | NS | | 1 |
| II | | | | | | 1.38 (1.05–1.82) |
| III | | | | | | 1.49 (1.01–2.70)* |
| Hormonal status | | | | | 6 | |
| Premenopausal | | | | | | 1 |
| Post-menopausal | | NS | | NS | | 1.27 (1.02–1.58) |

[a] Relative risks are referred to the best prognosis group. Esn, entry step number. ER, oestrogen receptor; Positivity if ⩾10 fmol $mg^{-1}$ cytosol protein. NS, not significant. *Not significant with the upper group.

*Evolution of the relative risk associated to ER status with elapsed time* As Figures 1 and 4 show a trend for the prognostic value of ER status to decrease with elapsed time, we have thus studied a model fitting this observation. For this purpose, we have introduced a dependence of time in the Cox model for the risk associated to ER status. This time-dependent model results in a better fitting of the data ($P<0.001$) than the proportional hazard model. Figures 7 and 8 represent the time-dependent evolution of the relative risks of developing metastases or of death according to ER status. The relative risk of death (4.68) for ER-negative tumours at diagnosis tends towards 1 after 8 years of follow-up. The relative risk of developing metastases linked to ER status decreases rapidly during the first 2 years.

*Prognostic value of ER and PR in low-risk patients* A total of 960 patients were older than 35 years with negative lymph nodes, a tumour size less than 50 mm and SBR grade I or II. The disease-free survival was shorter among patients with ER-negative tumours (RR = 1.34, $P=0.02$). The metastases-free survival was also shorter for ER-negative tumours (RR = 1.37, $P=0.02$). In both cases, PR status showed no prognostic value. The overall survival for these low-risk patients was found to be significantly different according to ER and PR status. The relative risks for ER-negative tumours was 1.62 ($P=0.006$) and 1.36 ($P=0.003$) for PR-negative tumours.

### Discussion

The present report is a multicentric study, with inherent advantages and pitfalls. Among the advantages are the large number of patients included in the study, the extensive follow-up and the absence of intervening adjuvant therapies. Conversely, since the data rely essentially upon the earliest steroid receptor assays performed for breast cancer management, one might question whether they are biased by a selection of large tumours because of technical requirements. As expected, when compared with recent studies displaying the pathological sizes with the same cut-off, a difference is observed in the proportions of T1 and T2 or more tumours. Our series include a smaller proportion of small tumours and more tumours over 20 mm than the recent ones (Mathiesen *et al.*, 1991; Stal *et al.*, 1992). Nevertheless, the observed survival rate for metastases-free patients in our series is similar to the statistics of the Fédération Nationale des Centres de Lutte Contre le Cancer (72% at 5 years and 53% at 10 years; Enquete Permanente Cancer, 1991).

Significant differences appeared between variable means among the different participating centres. Some of them can be ascribed to regional differences in patient and medical behaviour relating to the early diagnosis and management of breast cancer. For example, the series from centre number 2 shows the youngest and thus the highest premenopausal proportion of patients; the series from centre number 5 only includes node-negative patients since all the node-positive ones were discarded owing to adjuvant chemotherapy. Overall, it is conceivable that this heterogeneity may bring more statistical inferences than less dispersed series for the general population.

With regard to the data derived from laboratory work, two main points can lead to heterogeneous results: histological grading and receptor assays. Centre number 3 certainly shows the highest proportion of grade III tumours, but this is caused by a preselection of tumours of bad prognosis at the very beginning of the study. The observed variability among the proportion of the three histological grades from the different centres remains within the limits previously described in a multicentric study of SBR grading (Jacquemier *et al.*, 1981).

All steroid receptor assays were performed according to the same protocol, which was consequently published for the new groups intending to carry out the technique in their laboratories (Martin *et al.*, 1981). However, the percentage of ER-positive tumours varies widely between centres, probably owing to differences in technical skills in the beginning of receptor assays. But, allowing for their relatively small number of patients and the fact that receptor results were treated as discontinuous variables (positive/negative) in the statistical analysis, one might consider that their relative weight has thus been reduced.

Our statistical analysis of the optimal cut-off to separate positive and negative subgroups led to a value of 8 fmol $mg^{-1}$ cytosol protein for both receptors. For simplicity, we have preferred to keep the widely accepted cut-off of 10 fmol $mg^{-1}$ cytosol protein. A search for a pejorative cut-off in the elevated concentrations of ER and PR showed no significant differences up to 1000 fmol $mg^{-1}$ protein. Our series shows a better prognostic value for oestradiol receptor than for progesterone receptor in univariate analysis, in contrast with several previous studies (Thorpe *et al.*, 1987; Gelbfish *et al.*, 1988; Stal *et al.*, 1992), but the differences between the relative performances of the two receptors remain small nevertheless.

Multivariate analysis of the prognostic value of steroid receptors has been performed in 11 previous studies selected for their long follow-up and relatively large series of patients. Many of them addressed subgroups of patients, node-positive: Clark *et al.* (1993); node-negative: Thorpe *et al.* (1987), Silvestrini *et al.* (1995); patients under 50 years: Stal *et al.* (1992); infiltrating ductal carcinomas: Spyratos *et al.* (1989). Other studies presented results from unselected patients but with a mixed population receiving or not receiving systemic adjuvant therapy: Haybittle *et al.*, (1982), Chevallier *et al.* (1988), Aaltomaa *et al.* (1991), Gelbfish *et al.* (1988), Mathiesen *et al.* (1991). Only the study by Todd *et al.* (1987), based on the former patient population of Haybittle, displays results from an important group of primary breast cancers without adjuvant therapy and with a relatively long follow-up, however PR was not studied. The criteria introduced into the Cox regression model also vary: all the studies include the clinical or pathological size of the tumour and the degree of invaded lymph nodes. But, for other classical prognostic parameters, such as histological grade, only 7/11 of the studies have included the SBR or a form of histological classification (nuclear pleomorphism, mitotic index); the other studies comprised adjuvant therapies or proliferation criteria such as ploidy, S-phase or tritiated thymidine incorporation. Moreover, the varying number of criteria in the different Cox models, from four (Aaltomaa *et al.*, 1991) to ten (Chevallier *et al.*, 1988), may explain the diverse conclusions obtained. But, when both ER and PR were tested in the Cox model, a majority (7/9) of the studies concluded a better significance or a unique significance of PR and 3/11 showed a prognostic value for ER alone or for both receptors. It can be observed that the prognostic value of PR was particularly evidenced when the patients received adjuvant treatment.

The ER vanishing prognostic value, reaching a non-significant relative risk between negative and positive tumours during the follow-up, is difficult to analyse. A similar trend was previously described by Spyratos *et al.* (1989) in a study of 1262 patients from the Centre René Huguenin. Two kinds of explanation can be proposed: either this reflects intrinsic biological properties of the cancer cells evolving towards hormone resistance, or it is caused by intervening therapies used to control metastatic spreading.

The main conclusion that can be drawn from our study is that, by multivariate analysis, steroid receptor status has a relatively limited predictive value when compared with the well-established prognostic criteria of early breast cancer. Steroid receptor assays in breast tumours represent the very first step of a general strategy to decipher the biological behaviour of human breast cancer for clinical purposes. An array of biological prognostic factors (proliferation markers, growth factors, proteases, oncogenes . . .) has since been proposed. To this date, none of them has gained general acceptance for clinical practice. Steroid receptor status, although an imperfect predictor of patients' outcome, still remains the only single biological parameter in use to suggest therapeutic directives for subgroups of breast cancer patients.

### Participating centres

Institut Curie, Paris (Drs P Broet, H Magdelenat, B Asselain); Institut Gustave Roussy, Villejuif (Dr J-C Delarue); Centre René Huguenin, Saint-Cloud; (Drs M-F Pichon, F Spyratos), Centre Henri Becquerel, Rouen (Dr J-P Basuyau); Centre Léon Bérard, Lyon (Dr S Saez); Institut Jean Godinot, Reims (Dr A Rallet); Centre Claudius Régaud, Toulouse (Pr P Courriere); Centre Paul Strauss, Strasbourg (Dr R Millon).

**Acknowledgements**
The initial work on receptor assays for the Centre René Huguenin series was previously performed by Dr MF Pichon in the Laboratoire de Biochimie Hormonale, Faculté de Médecine Paris-Sud (Pr E Milgrom). Dr P Broet is supported by a fellowship from the 'Fond d'Études et de Recherche du Corps Médical des Hopitaux de Paris'.

### References

AALTOMAA S, LIPPONEN P, ESKILINEN M, KOSMA VM, MARIN S, ALHAVA E AND SYRJANEN K. (1991). Hormone receptors as prognostic factors in female breast cancer. *Ann. Med.*, **23**, 643–648.

ADAMI HO, MALKER B, HOLMBERG L, PERSONN I AND STONE B. (1986). The relation between survival and age at diagnosis in breast cancer. *N. Engl. J. Med.*, **315**, 559–563.

BLOOM HJG AND RICHARDSON WW. (1957). Histological grading and prognosis in breast cancer. A study of 1409 cases of which 359 have been followed 15 years. *Br. J. Cancer*, **11**, 359–377.

CHEVALLIER B, HEINZMANN F, MOSSERI V, DAUCE JP, BASTIT P, GRAIC Y, BRUNELLE P, BASUYAU JP, COMOZ M AND ASSELAIN B. (1988). Prognostic value of estrogen and progesterone receptors in operable breast cancer. *Cancer*, **62**, 2517–2524.

CLARK GM, WENGER CR, BEARDSLEE S, OWENS MA, POUNDS G, OLDAKER T, VENDRELY P, PANDIAN MR, HARRINGTON D AND McGUIRE WL. (1993). How to integrate steroid hormone receptor, flow cytometric, and other prognostic information in regard to primary breast cancer. *Cancer*, **71**, 2157–2162.

COX DR. (1972). Regression models and life table (with discussion). *J. R. Stat. Soc. B*, **34**, 187–202.

ENQUETE PERMANENTE CANCER 1975–1986. (1991). In *Monographie des Cancers du Sein*. Féderation Nationale des Centres de Lutte contre le Cancer (FNCLCC). pp. 37. Doin: Paris.

GELBFISH GA, DAVIDSON AL, KOPEL S, SCHREIBMAN B, GELBFISH JS, DEGENSHEIN GA, HERZ BL AND CUNNINGHAM JN. (1988). Relationship of estrogen and progesterone receptors to prognosis in breast cancer. *Ann. Surg.*, **207**, 75–79.

HAYBITTLE JL, BLAMEY RW, ELSTON CW, JOHNSON J, DOYLE PJ, CAMPBELL FC, NICHOLSON RI AND GRIFFITHS K. (1982). A prognostic index in primary breast cancer. *Br. J. Cancer*, **45**, 361–366.

HOST H AND LUND E. (1986). Age as a prognostic factor in breast cancer. *Cancer*, **57**, 2217–2221.

JACQUEMIER J, VAGUE D, LIEUTAUD R, DE MASCAREL I, TROJANI M, MEUGE C, LE DOUSSAL V, GENTILE A, HEBERT H, KEMENY F, AMOUROUX J AND CONTESSO G. (1981). Définition et reproductibilité du grading de Scarff et Bloom. In *Evaluation des Moyens de Diagnostic du Cancer du Sein*, Gest J. (ed.) pp. 173–185. JMT Conseil: Paris.

KALBFLEISH JD AND PRENTICE RL. (1980). The statistical analysis of failure time data. pp. 122–127. John Wiley and Sons: New York.

KAPLAN EL AND MEIER P. (1958). Non-parametric estimation for incomplete observation. *J. Am. Stat. Assoc.*, **53**, 457–471.

MARTIN PM, BRESSOT N, DELARUE JC, MAGDELENAT H, PICHON MF, SAEZ S AND SPYRATOS F. (1981). Protocole coopératif intercentres. Dosage des récepteurs hormonaux stéroidiens intratissulaires en pathologie mammaire humaine. In *Evaluation des Moyens de Diagnostic du Cancer du Sein*, Gest J. (ed.) pp. 173–185. JMT Conseil: Paris.

MATHIESEN O, BONDERUP O, CARL J, PANDURO J AND PEDERSEN KO. (1991). The prognostic value of estrogen and progesterone receptors in female breast cancer. *Acta Oncol.*, **30**, 691–695.

PETO R AND PETO J. (1972). Asymptotically efficient rank invariant test procedures (with discussion). *J.R. Stat. Soc. A*, **135**, 185–207.

RAEMAEKERS JMM, BEEX LVAM, KOENDERS AJM, PIETERS GFFM, SMALS AGH, BENRAAD TJ, KLOPPENBORG PWC AND THE BREAST CANCER GROUP. (1985). Disease-free interval and estrogen receptor activity in tumor tissue of patients with primary breast cancer: analysis after long-term follow-up. *Breast Cancer Res. Treat.*, **6**, 123–130.

SILVESTRINI R, DAIDONE MG, LUISI A, BORACCHI P, MEZZETI M, DI FRONZO G, ANDREOLA S, SALVADORI B AND VERONESI U. (1995). Biologic and clinicopathologic factors as indicators of specific relapse types in node-negative breast cancer. *J. Clin. Oncol.*, **13**, 697–704.

SPYRATOS F, HACENE K, TUBIANA-HULIN M, PALLUD C AND BRUNET M. (1989). Prognostic value of estrogen and progesterone receptors in primary infiltrating ductal breast cancer. *Eur J. Cancer Clin. Oncol.*, **25**, 1233–1240.

STAL O, CARSTENSEN J, HATSCHEK T AND NORDENKJOLD B. (1992). Significance of S-phase fraction and hormone receptor content in the management of young breast cancer patients. *Br. J. Cancer*, **66**, 706–711.

THORPE SM, ROSE C, RASMUSSEN BB, MOURIDSEN HT, BAYER T AND KEIDING N. (1987). Prognostic value of steroid hormone receptors: multivariate analysis of systematically untreated patients with node negative primary breast cancer. *Cancer Res.*, **47**, 6126–6133.

TODD JH, DOWLE C, WILLIAMS MR, ELSTON CW, ELLIS IO, HINTON CP, BLAMEY RW AND HAYBITTLE JL. (1987). Confirmation of a prognostic index in primary breast cancer. *Br. J. Cancer*, **56**, 489–492.


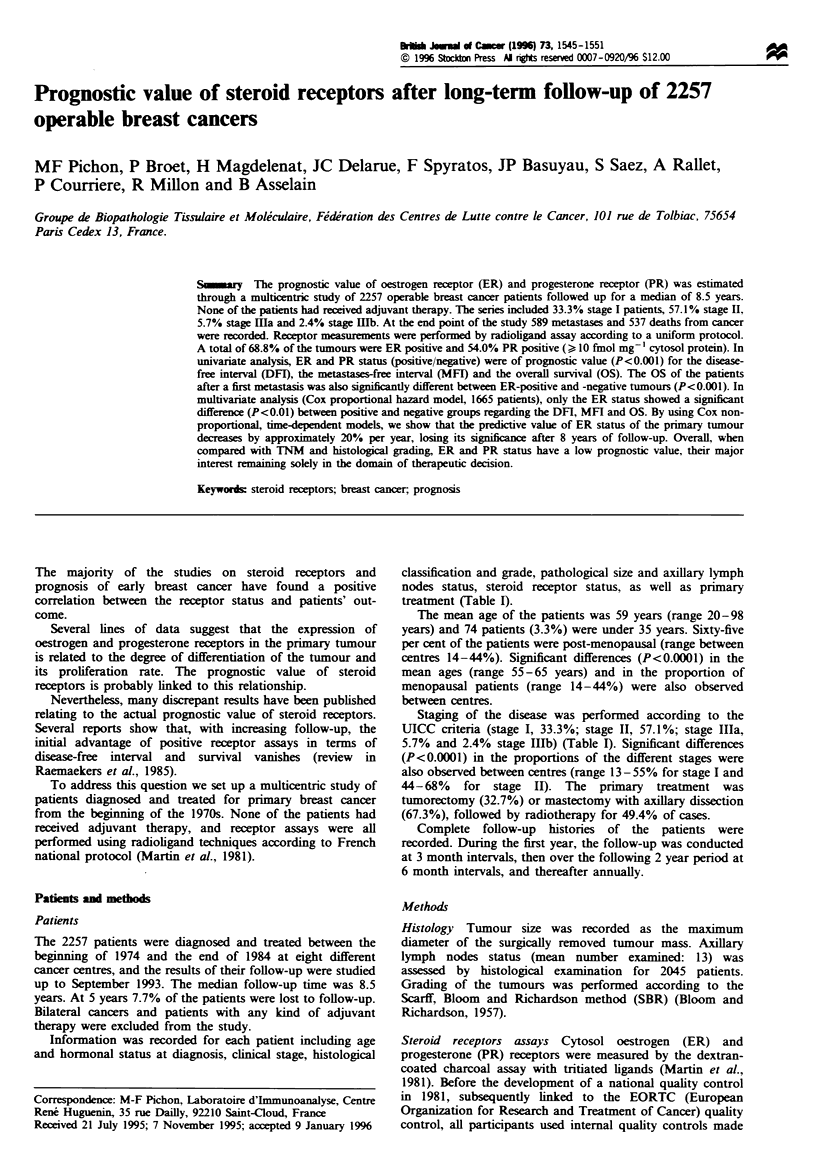

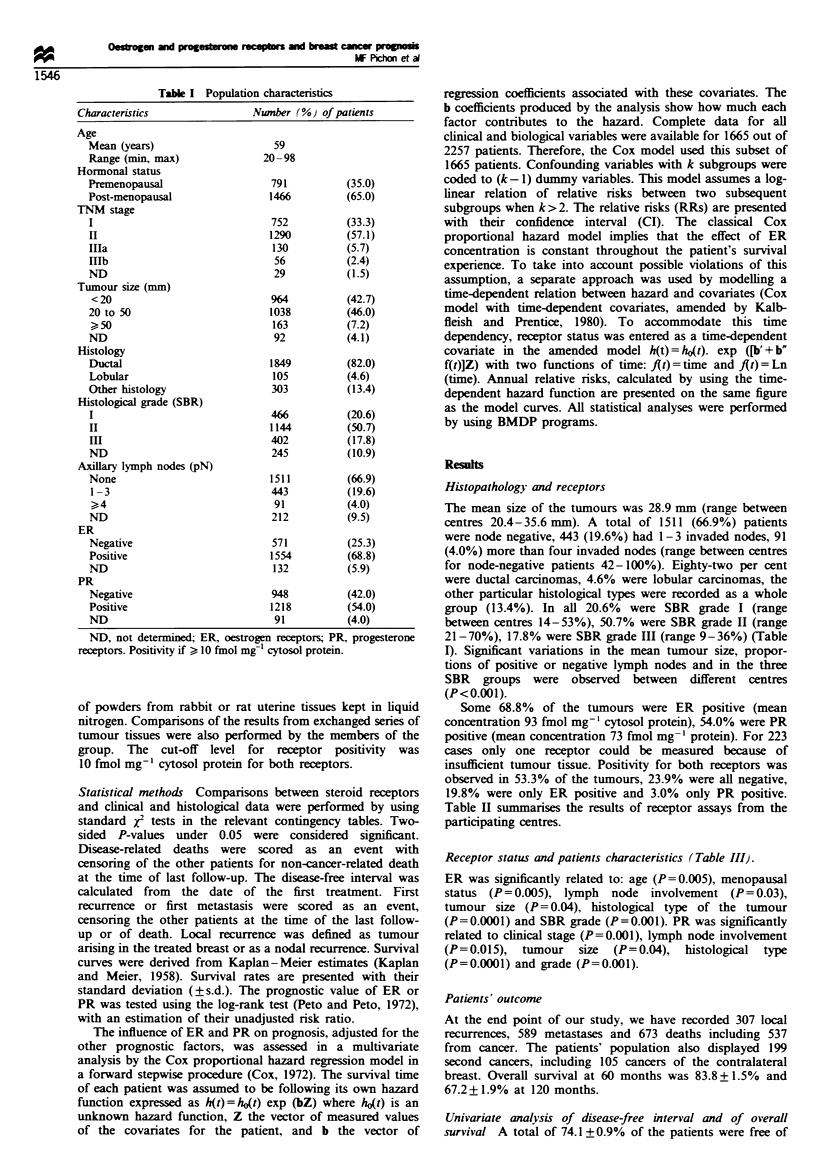

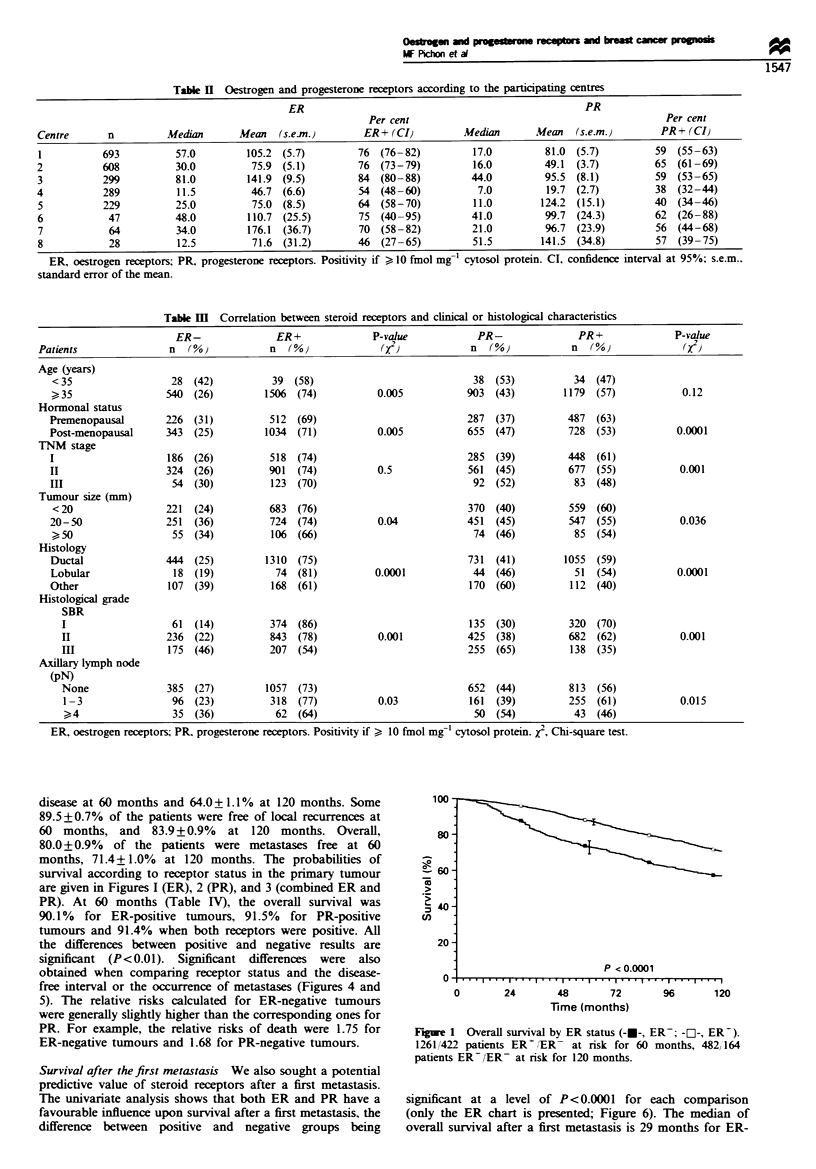

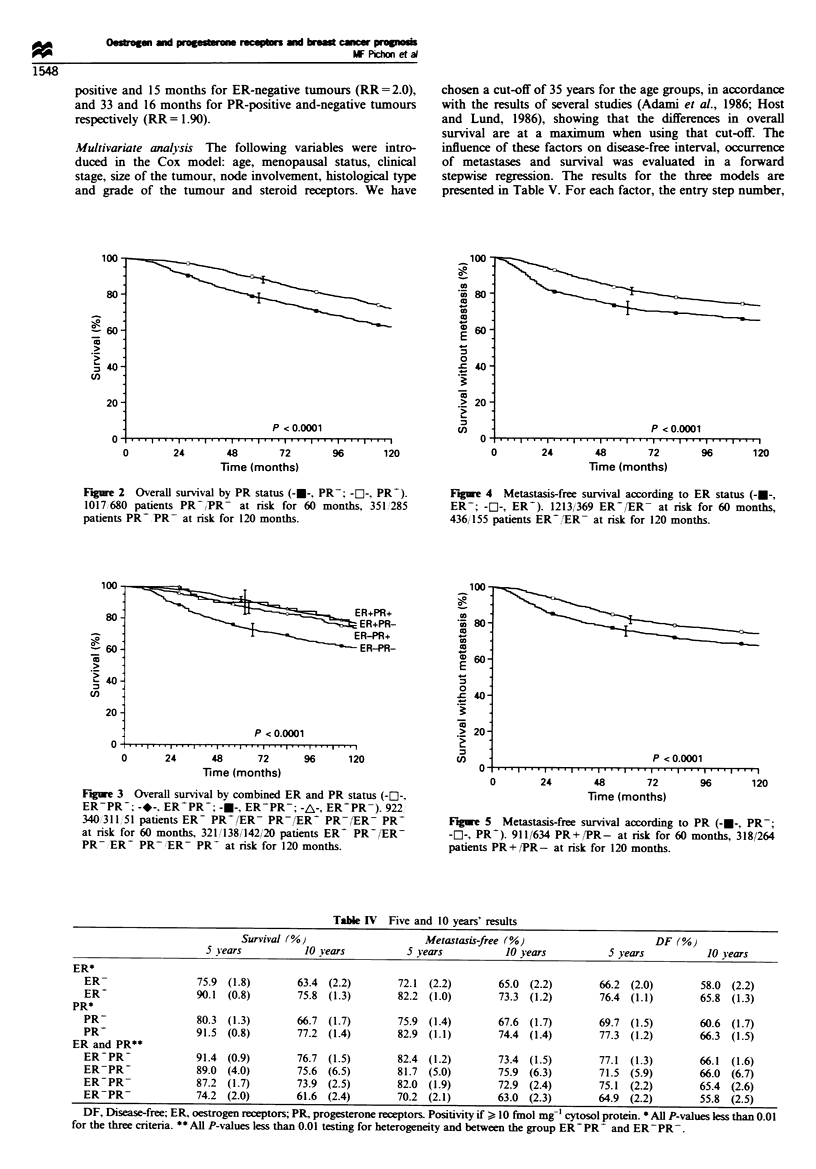

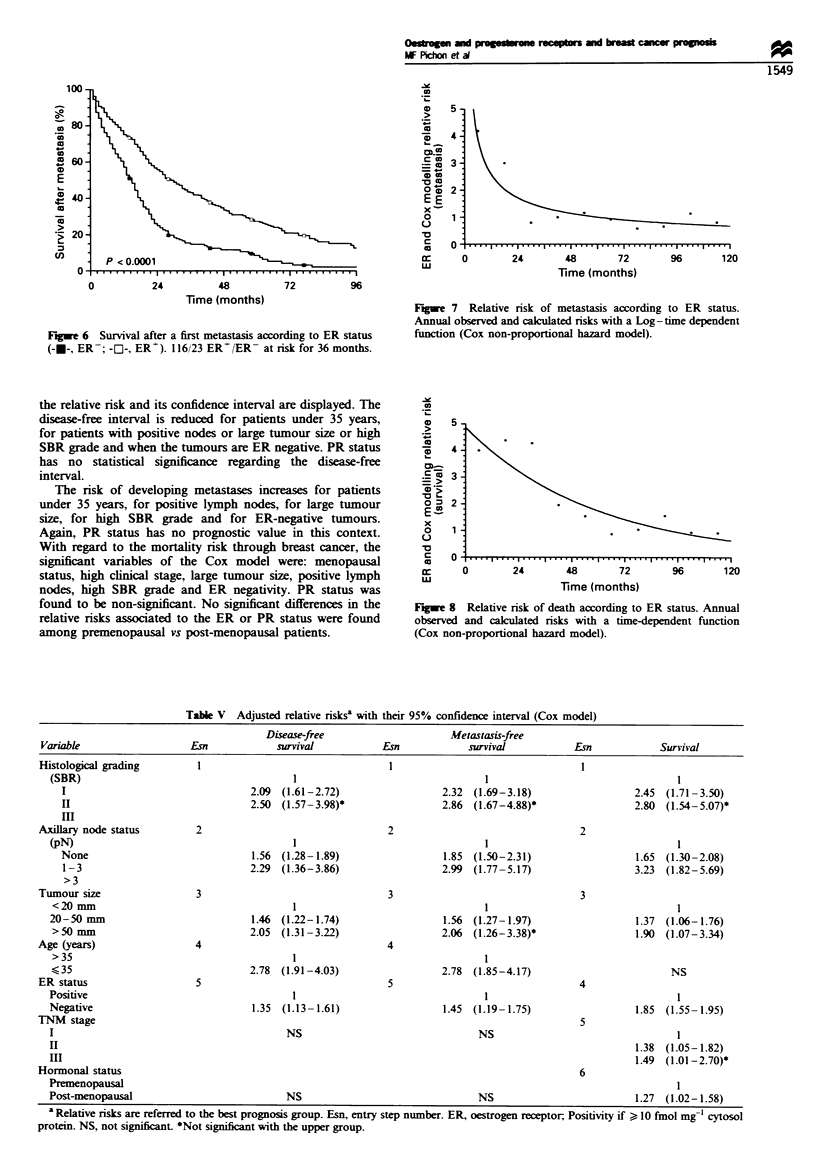

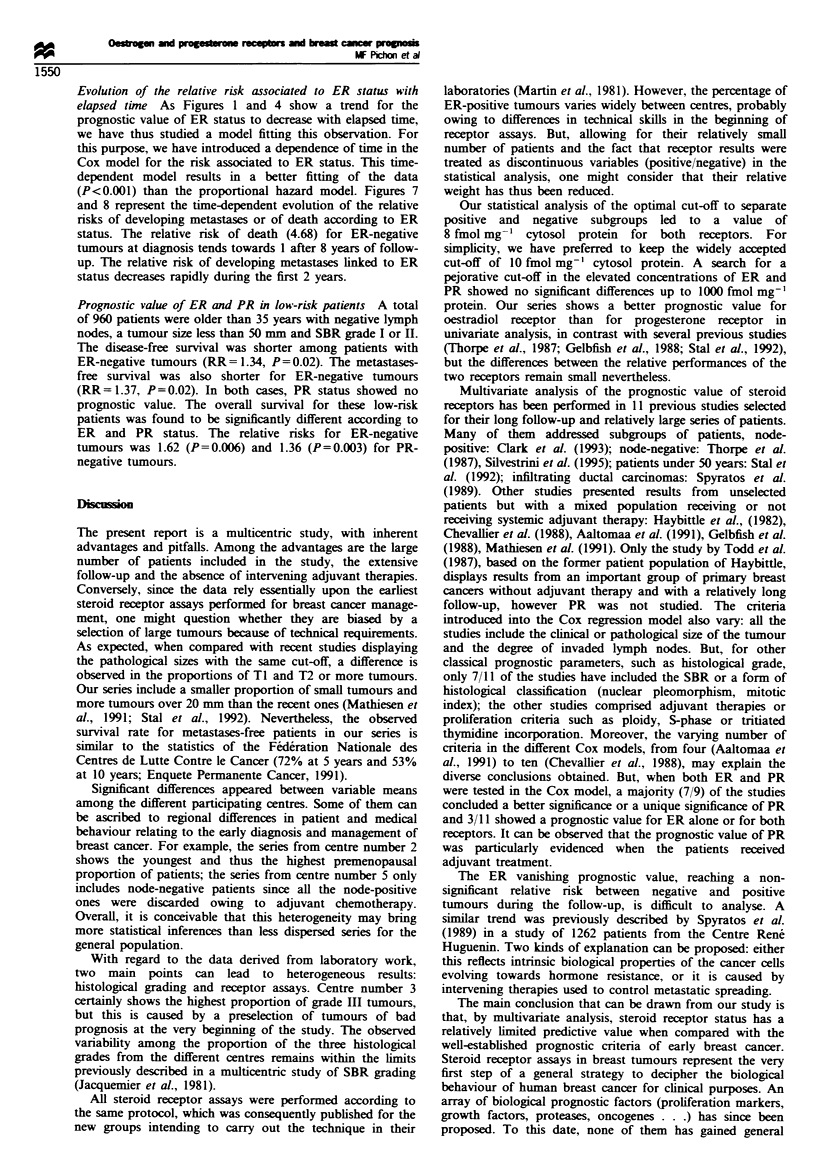

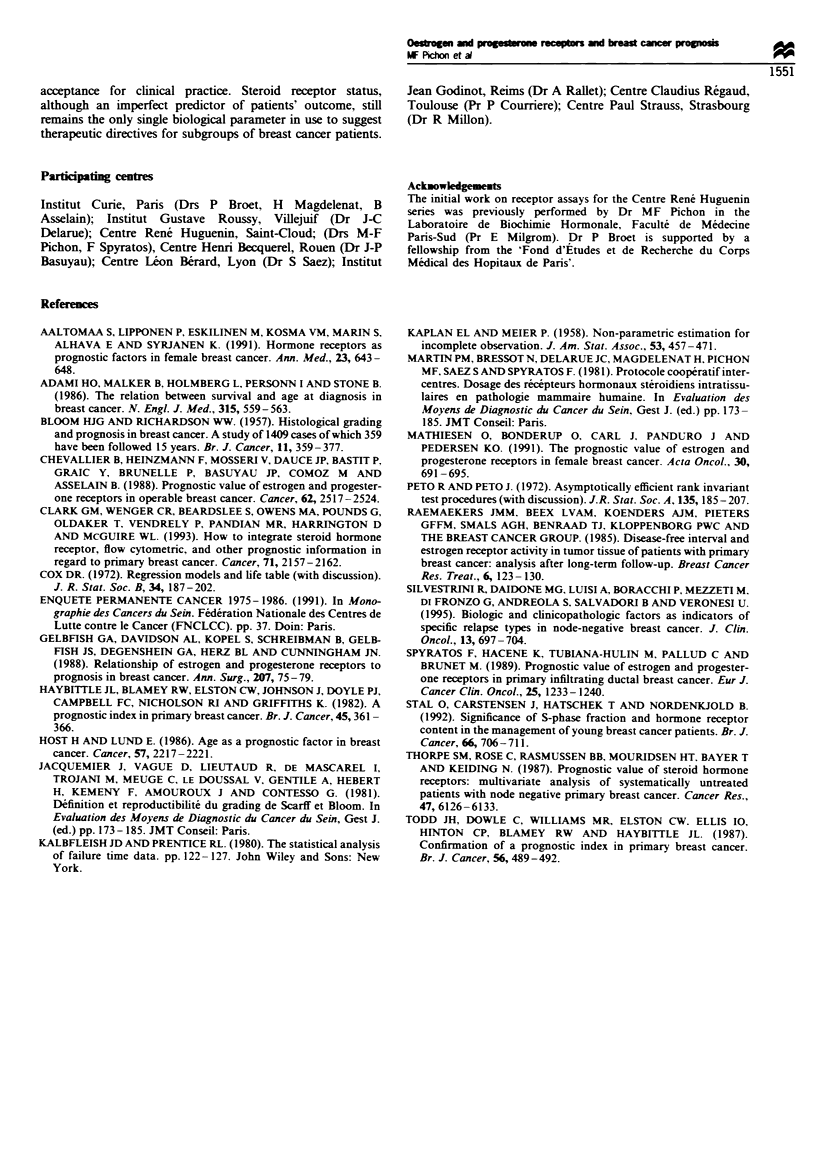


## References

[OCR_01030] Aaltomaa S., Lipponen P., Eskelinen M., Kosma V. M., Marin S., Alhava E., Syrjänen K. (1991). Hormone receptors as prognostic factors in female breast cancer.. Ann Med.

[OCR_01038] Adami H. O., Malker B., Holmberg L., Persson I., Stone B. (1986). The relation between survival and age at diagnosis in breast cancer.. N Engl J Med.

[OCR_01043] BLOOM H. J., RICHARDSON W. W. (1957). Histological grading and prognosis in breast cancer; a study of 1409 cases of which 359 have been followed for 15 years.. Br J Cancer.

[OCR_01046] Chevallier B., Heintzmann F., Mosseri V., Dauce J. P., Bastit P., Graic Y., Brunelle P., Basuyau J. P., Comoz M., Asselain B. (1988). Prognostic value of estrogen and progesterone receptors in operable breast cancer. Results of a univariate and multivariate analysis.. Cancer.

[OCR_01051] Clark G. M., Wenger C. R., Beardslee S., Owens M. A., Pounds G., Oldaker T., Vendely P., Pandian M. R., Harrington D., McGuire W. L. (1993). How to integrate steroid hormone receptor, flow cytometric, and other prognostic information in regard to primary breast cancer.. Cancer.

[OCR_01067] Gelbfish G. A., Davidson A. L., Kopel S., Schreibman B., Gelbfish J. S., Degenshein G. A., Herz B. L., Cunningham J. N. (1988). Relationship of estrogen and progesterone receptors to prognosis in breast cancer.. Ann Surg.

[OCR_01075] Haybittle J. L., Blamey R. W., Elston C. W., Johnson J., Doyle P. J., Campbell F. C., Nicholson R. I., Griffiths K. (1982). A prognostic index in primary breast cancer.. Br J Cancer.

[OCR_01081] Høst H., Lund E. (1986). Age as a prognostic factor in breast cancer.. Cancer.

[OCR_01111] Mathiesen O., Bonderup O., Carl J., Panduro J., Pedersen K. O. (1991). The prognostic value of estrogen and progesterone receptors in female breast cancer. A single center study.. Acta Oncol.

[OCR_01120] Raemaekers J. M., Beex L. V., Koenders A. J., Pieters G. F., Smals A. G., Benraad T. J., Kloppenborg P. W. (1985). Disease-free interval and estrogen receptor activity in tumor tissue of patients with primary breast cancer: analysis after long-term follow-up.. Breast Cancer Res Treat.

[OCR_01128] Silvestrini R., Daidone M. G., Luisi A., Boracchi P., Mezzetti M., Di Fronzo G., Andreola S., Salvadori B., Veronesi U. (1995). Biologic and clinicopathologic factors as indicators of specific relapse types in node-negative breast cancer.. J Clin Oncol.

[OCR_01135] Spyratos F., Hacene K., Tubiana-Hulin M., Pallud C., Brunet M. (1989). Prognostic value of estrogen and progesterone receptors in primary infiltrating ductal breast cancer. A sequential multivariate analysis of 1262 patients.. Eur J Cancer Clin Oncol.

[OCR_01140] Stål O., Carstensen J., Hatschek T., Nordenskjöld B. (1992). Significance of S-phase fraction and hormone receptor content in the management of young breast cancer patients.. Br J Cancer.

[OCR_01144] Thorpe S. M., Rose C., Rasmussen B. B., Mouridsen H. T., Bayer T., Keiding N. (1987). Prognostic value of steroid hormone receptors: multivariate analysis of systemically untreated patients with node negative primary breast cancer.. Cancer Res.

[OCR_01151] Todd J. H., Dowle C., Williams M. R., Elston C. W., Ellis I. O., Hinton C. P., Blamey R. W., Haybittle J. L. (1987). Confirmation of a prognostic index in primary breast cancer.. Br J Cancer.

